# Effect of childhood BMI on asthma: a systematic review and meta-analysis of case-control studies

**DOI:** 10.1186/s12887-018-1093-z

**Published:** 2018-04-26

**Authors:** Yosra Azizpour, Ali Delpisheh, Zahra Montazeri, Kourosh Sayehmiri, Behzad Darabi

**Affiliations:** 1 Department of Clinical Epidemiology, Student Research Committee, Ilam University of Medical Sciences, Ilam, Iran; 2 Department of Clinical Epidemiology, Psychosocial Injuries Research Center, Ilam University of Medical Sciences, Ilam, Iran; 3 School of Epidemiology, Public Health and Preventive Medicine, Faculty of Medicine, University of Ottawa, Ottawa, Canada; 4 Department of Biostatistics, Psychosocial Injuries Research Center, Ilam University of Medical Sciences, Ilam, Iran; 5 Department of Pediatrics, Faculty of Medicine, Ilam University of Medical Sciences, Ilam, Iran

**Keywords:** Asthma, Adolescences, Body mass index, Childhood, Meta-analysis

## Abstract

**Background:**

Asthma is a multifactorial syndrome that threatens the health of children. Body mass index (BMI) might be one of the potential factors but the evidence is controversial. The aim of this study is to perform a comprehensive meta-analysis to investigate the association between asthma and BMI.

**Methods:**

Electronic databases including, Web of Science, Pubmed, Scopus, Science Direct, ProQuest, up to April 2017, were searched by two researchers independently. The keywords “asthma, body mass index, obesity, overweight, childhood and adolescence” were used. Random and fixed effects models were applied to obtain the overall odds ratios (ORs) and standardized mean difference (SMD). Heterogeneity between the studies was examined using I^2^ and Cochrane Q statistics.

**Results:**

After reviewing 2511 articles, 16 studies were eligible for meta-analysis according to inclusion/exclusion criteria. A meta-analysis from 11 case-control studies revealed OR of asthma and overweight as OR = 1.64; (95% Confidence Interval (CI): 1.13–2.38) and from 14 case-control studies, OR for asthma and obesity was OR = 1.92 (95% CI: 1.39–2.65), which indicated that risk of asthma in overweight and obese children and adolescence was significantly higher (1.64 and 1.92 times) than that of individuals with (*p*-value < 0.01 for underweight/normal weight in both cases). Furthermore, there was a significant relationship between asthma and BMI > 85 percentile according to SMD SMD = 0.21; (95%CI: 0.03–0.38; p-value = 0.021).

**Conclusions:**

The results showed a significant relationship between BMI (obesity/overweight) and asthma among children and adolescents. It is important to study the confounding factors that affect the relationship between asthma and BMI in future epidemiological researches.

**Electronic supplementary material:**

The online version of this article (10.1186/s12887-018-1093-z) contains supplementary material, which is available to authorized users.

## Background

There are some hypotheses for the relationship between asthma and obesity since the number of the cases diagnosed with these two disorders over the last two decades has increased [1]. Asthma is a chronic clinical respiratory syndrome that is accompanied by the inflammation of respiratory ducts, obstruction, and airway hyper responsiveness [2]. It is caused by a combination of factors and complicated interaction between hereditary traits, air pollution, respiratory tract infection, and exposure to triggers such as cigarette smoking [3]. These factors influence the response of the disease to treatment and its severity [4]. It is estimated that 7.1 million individuals under 18 years of age were currently afflicted with asthma and 4.1 million suffered from periodic asthma or asthma attack in 2011 (United States) [5]. Over the last three decades, prevalence of obesity has doubled and quadrupled among children and adolescents [6, 7] and along with other mechanisms, obesity may cause shortness of breath as well. It is known that aggregation of soft fatty tissues around the chest increases pressure on the lungs, increases blood volume at the area, and consequently, decreases the capacity of the respiratory system. Furthermore, other mechanical effects of obesity may cause limitations to airways and hypersensitivity [1]. So, lack of enough physical activity among asthma patients and physiological respiratory changes of obese patients may cause the two diseases to be interrelated [8].

There are four previous meta-analyses conducted by Chen [9], Flaherman [10], Egan [11] and Mebrahtu [12] in which Relative Risk (RR) or Odds Ratio (OR) for relationships between asthma and overweight among children were reported. Three of these meta-analyses are based on cohorts (Chen, Egan and Flaherman) and the other one is based on any observational studies including cohort, case-control, and cross-sectional. Chen and Egan applied subgroup analysis just for gender but they didn’t conduct a cumulative meta-analysis. Flaherman considered studies that reported both high birth weight and high BMI in school aged children for cumulative meta-analysis and they reported OR and RR and applied subgroup analysis for physician diagnosis. Mebrahtu was more consistent in investigating this association by determining OR in different weight categories. We applied an intensive search and employed comprehensive analyses not only based on OR estimates, but also we considered SMD analysis, cumulative meta-analysis and adjusted ORs. Subgroup analyses for gender, age, continents, and asthma diagnosis method, year of publication and sample size were applied. In addition, case-control studies have been considered for risk ratio assessment.

## Methods

This systematic review was based on Preferred Reporting Items for Systematic Reviews and Meta-Analyses (PRISMA) guidelines [13] (Additional file 1: PRISMA Checklist S1).

### Criteria of research

All case-control studies on the relationship between BMI and asthma among childhood and adolescence regardless of time and place of study were considered, language was limited to English.

### Search strategy

A comprehensive search was undertaken via Web of Science (1983 to 10 April of 2017), PubMed /Medline (1966 to 10 April of 2017), Scopus (1960 to 10 April of 2017), Science Direct (1823 to 10 April of 2017), ProQuest (1993 to 10 April of 2017), Google Scholar (web search engine), and Eastern Mediterranean Region databases (IMEMR) (1984 to 10 April of 2017). Medical subject headings (MeSH) keywords such as “asthma, BMI, obesity, overweight, childhood and adolescence” were used for our search in scientific journals, conferences, dissertations, theses and reports. All references to relevant articles (manually) were also investigated.

For example, the following box represents the search strategies in PubMed

1. “Asthma” [MeSH]
2. Childhood [MeSH]
3. Adolescence [MeSH]
4. #1 AND #2 AND #3
5. “Body mass Index” [MeSH]
6. #4 AND #5
7. “Obesity” [MeSH]
8. #4 AND #7
9. “Overweight” [MeSH]
10. #4 AND #9

### Asthma diagnosis

The case group (asthmatic) was diagnosed either by a physician or by completion of the ISSAC (The International Study of Asthma and Allergies in Childhood) questionnaire by a parent or adolescent. The control group (non-asthmatic) consisted of those who were not diagnosed with asthma.

### BMI criteria

The following criteria were considered in assessing the exposure factor (BMI): 1. Age-sex-specific BMI percentiles were obtained based on Centers for Disease Control and Prevention (CDC) growth chart (see Table 1). 2. Age-sex-specific cut-off points (underweight18.5 kg/m^2^, overweight: 25 kg/m^2^ and obesity 30 kg/m^2^) by the International Obesity Task Force (IOTF) [14]. 3. Reference data for obesity with normal being < 85 ^th^, obese > 85 ^th^ - <95th and very obese ≥95 ^th^ [15]. 4. The BMI percentile values with underweight being ≤P5, Malnutrition >P5- ≤ P15, normal >P15- < P85, overweight ≥P85- < P95, obese ≥P95 [16]. 5. The BMI-Z score based on CDC growth chart. 6. The overweight/obesity when the BMI- standard deviation score (SDS) units (z-score) was ≥2 [17]. (It is notable that we used BMI percentiles based on the CDC growth chart (2014) where underweight < 5th, normal ≥5th- < 85th, overweight≥85th- < 95th and obese ≥95th [18] and the categorization method of the IOTF for exposure (BMI)).

**Table 1:** Case-control studies included in the research

Author	Sample	country	Exposure categorization method	Age group (year)	Asthma Diagnosis	Publication year	Time duration	Quality Score
Gennuso & et al. [48]	Case:85	(USA)	Reference data Normal <85, Obese: ≥ 85^th^ <95^th^.Very obese: ≥ 95^th^ [15]	Case: 4-16	Physician diagnosed asthma	1998	N/R	80
	Control:86			Control:4-16				
Vignolo & et al. [49]	Case:554	(Italy)	BMI- (SDS) units (z-score)	Case: 2.2-16.1	Physician diagnosed asthma	2005	Case: January –December 2001	80
	Control:625			Control: 2.4–16.3			Control: April-May 2001	
Mansell & et al. [50]	Case:134	(USA)	Overweight >85^th^ and non overweight <85^th^ (CDC growth charts)	Case: 12-18	Physician diagnosed asthma	2006	N/R	80
	Control:82			Control:12-18				
Vargas & et al. [51]	Case:213	(USA)	Risk for overweight ≥ 85^th^-<95^th^/ overweight ≥ 95^th^ (CDC growth charts )	Case: 3-5	Physician diagnosed asthma	2007	November 2000-December 2003	90
	Control:816			Control:3-5				
Careneiro bertolace & et al. [52]	Case:231	(Brazil)	Overweight :≥ 85^th^ and Obese: ≥ 95^th^ (CDC growth charts)	Case: 13-14	Completed ISAAC questionnaire by adolescence	2008	March-December 2005	80
	Control:190			Control:13-14				
Henkin & et al. [40]	Case:94	(USA) (Asian patient)	Underweight<20%, normal 20-85%, risk for overweight 85-95% & Overweight >95% (CDC growth charts	Case: 4-18	Physician-diagnosed asthma	2008	N/R	100
	Control:94			Control:4-18				
Walders-Abramson & et al. [53]	Case:59	(USA)	≥95^th^ obese,” 85^th^–95^th^ “overweight,”15 and <85^th^ “normal weight (CDC growth charts)	Case: 10-16	Physician-diagnosed asthma	2009	December 2005-July 2007	80
	Control:59			Control:10-16				
Tsai & et al. [54]	Case:27	(USA)	Age- and sex-specific cut-off points for childhood by (IOTF)	Case: 9-11	Parent reported according physician-diagnosed asthma and meds use	2012	2004-2006	80
	Control:27			Control:9-11				
Ahmadi-afshar & et al. [55]	Case:200	(Iran)	Overweight : BMI>85^th^ and Obese BMI>95^th^ (CDC growth charts)	Case: 6-15	Physician-diagnosed asthma	2013	March-September 2010	80
	Control:200			Control:6-15				
Scepanovic & et al. [16]	Case:354	(Montenegro)	Based on the BMI percentile values	Case: 6-15	Physician-diagnosed asthma	2013	N/R	70
	Control:354			Control: 6-15				
Nahhas & et al. [43]	Case:632	(Saudi Arabia)	Underweight <5^th^, Normal 5^th^-85^th^, Overweight 85^th^-94^th^ & obese ≥ 95 (CDC growth charts)	Case: 6-8	Complete a ISAAC questionnaire by parent	2014	N/R	90
	Control:632			Control: 6-8				
Forno & et al. [42]	Case:351	(Puerto Rico)	BMI z scores based on CDC growth charts	Case: 6-14	Physician-diagnosed asthma	2014	March 2009 -June 2010	90
	Control:327			Control: 6-14				
Papoutsakis & et al. [41]	Case:217	(Greece )	Age- and sex-specific cut-off points for childhood by (IOTF)	Case: 5-11	Physician-diagnosed asthma	2015	November 2007-September 2010	90
	Control:297			Control: 5-11				
Rice & et al. [56]	Case:287	(Peru)	Age- and sex-specific cut-off points for childhood by (IOTF)	Case: 9-19	Physician-diagnosed asthma	2015	N/R	80
	Control:96			Control: 9-19				
Groth & et al. [57]	Case:61	(USA)	Underweight <5^th^, Normal ≥ 5^th^ and < 85^th^, Overweight ≥ 85^th^ and < 95^th^ obese ≥ 95^th^ (CDC growth charts)	Case: 12-15	Physician-diagnosed asthma	2016	2002-2004	90
	Control:484			Control: 12-15				
Lawson& et al. [44]	Case:78	(Canada)	Age- and sex-specific cut-off points for childhood by (IOTF)	Case: 6-14	Physician-diagnosed asthma	2017	2011	100
	Control:451			Control: 6-14				

*N/R* Not reported

### Article selection

Searching databases using keywords and extracting data from articles were done independently by two researchers (Azizpour and Sayehmiri) in order to avoid risk of bias. An abstract of each article was screened for eligibility according to inclusion/exclusion criteria and then the full text was reviewed for data extraction. In cases of disagreement between the two reviewers, a third researcher reviewed the article and a final decision was made after careful discussion. The relevant articles were selected according to inclusion/exclusion criteria. Inclusion criteria were case-control studies on the relationship between asthma and BMI, studies on children and adolescents (2–19 years old), and in the English language. Studies on adults, irrelevance of the subject, the relationship between BMI and asthma severity, cross-sectional and cohort studies and any relationship between breast feeding and asthma were excluded. For the quality assessment of studies we used, the Joanna Briggs Institute (JBI) Critical Appraisal Tools [19]. The quality score is determined by the range 67–100 (good), 34–66 (average), and 0–33 (bad).

### Data extraction

An appropriate data extraction form was designed including author (s) name, sample size, country of study, age group (case and control), method of asthma diagnosis, year of publication, time of study, and the exposure assessment method (BMI). The following data, if available, are extracted to evaluate the association between BMI and asthma.

1. Frequency of obesity, overweight and underweight/normal in childhood and adolescence (based on CDC growth chart (2014), and IOTF) in both case and control groups (binary outcome) to obtain OR and RR.
2. Mean and standard deviation (M ± SD) of BMI based on BMI or BMI-Z score in case and control groups (continuous measure) to obtain SMD.
3. Adjusted OR to evaluate the association between BMI and asthma.
4. The above data were also collected for different gender, age, continents, and asthma diagnosis method, year of publication and sample size for the purpose of subgroup analyses.

### Statistical analyses

The following three methods were employed to aggregate the extracted data and drive the summary effect of an association between asthma and BMI:

Method 1: OR and RR were calculated when classified BMI (overweight/obesity) for control and case groups were reported (${OR}_i=\frac{a_i{d}_i}{b{{}_ic}_i}$,$RR=\frac{a/\left(a+b\right)}{c/\left(c+d\right)}$), then Der Simonian and Laird method (random effects model) were used to combine the OR_S_ or RR_S_.

Cumulative meta-analysis was used for pooled estimates to show whether the year of publication or year of the new study has any essential effect on the final results.

Method 2: To derive the summary effects for studies that reported the effect size for case and control groups based on the mean and standard deviation (SD) of BMI, or BMI-Z score, we first calculated the mean difference of each study as follows:

$$ SMD=\frac{\overline {X_1}-\overline {X_2}}{S_{pooled}} $$

where

SMD: standardized mean difference.

SD: pooled standard deviation

$$ {S}_{Pooled}^2=\frac{\left({n}_1-1\right){S}_1^2+\left({n}_2-1\right){S}_2^2}{n_1+{n}_2-2} $$

S_1_^2^: Variance of the case group

S_2_^2^: Variance of the control group

*n*_1_: Number of samples in the case group

*n*_2_: Number of samples in the control group

Afterwards, to find the overall SMD of all articles, Der Simonian and Laird method (random effects model) was used to combine the result of studies with the “metan” command (from STATA).

Method 3: Ln transformation, Ln (_OR_) of each adjusted OR and associated confidence interval was calculated by using the following formula for standard error:

$$ SE=\frac{\ln \left(\frac{OR_{Upper}}{OR_{Lower}}\right)}{2\times 1.96} $$

where *OR*_*Upper*_ is upper limit of OR and *OR*_*Lower*_ is lower limit of OR [20].

Then the inverse variance method (fixed effect model) was used to obtain the overall effect size (ES), and the anti-log of the overall ES was taken to come back to the original OR, (*e*^*ln (OR)*^).

Subgroups analyses were performed for gender, age, continent, sample size, year of publication and asthma diagnosis methods.

In addition, I^2^ and Cochrane Q Statistics were used [21] to investigate the heterogeneity of the data. I^2^ was considered in four levels; I^2^ = 0% indicated no heterogeneity, 25% to − 50% for low, 50% to 75% for moderate, and more than 75% for high heterogeneity [22]. A random or fixed model was used according to the heterogeneity factor whereas random effects models [23, 24] were used for heterogeneous studies and fixed effects models otherwise.

Begg’s test was used to check publication bias. All data analyses were performed in STATA version 10 and a *p*-value < 0.05 was considered as statistically significant.

## Results

### Literature search and data collection

**Fig. 1:**
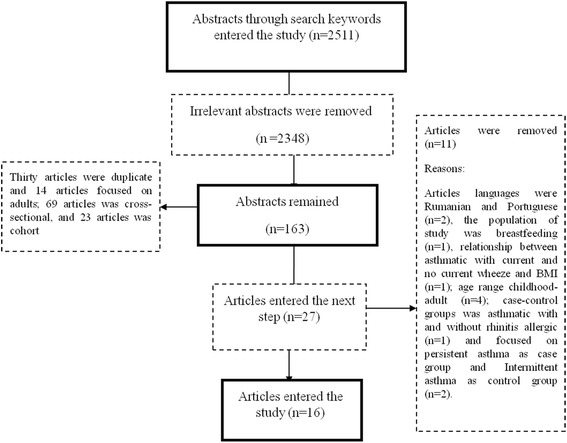
Flowchart of selecting the article

In total, 2511 titles were found from which 2348 were removed after reviewing abstracts. Out of 163 potentially related articles, 30 were duplicates and 14 were removed since the ages of the participants were out of our scope. In addition, 69 cross-sectional and 23 cohort studies were also removed. Afterwards, out of 27 articles, 11 case-control studies were removed for the following reasons: Two articles were not in English (One in Romanian [25] and the other in Portuguese [26]), the population of one study was breastfeeding [27], one article studied the relationship between asthmatic with current and no current wheeze and BMI [28], four studies were on age range childhood-adult [29–32], one in which case-control groups were asthmatic with and without allergic rhinitis [33], and finally two studies focused on persistent asthma as the case group and Intermittent asthma as the control group [34, 35] (2001 to 2016). Finally, 16 case-control articles from 1998 to 2017 were entered into the study (Fig. 1).

Overall, 9 studies in the United States of America and Canada (North America), 2 in Peru and Brazil (South America), 3 in Montenegro, Greece and Italy (Europe) and 2 in Iran and Saudi Arabia (Asia) were identified with an age range of 2.2–18 years of age in case group and 2.4–18 years of age in control groups; in total 3577 individuals were in the case group and 4820 individuals were in the control group. In 13 studies, asthma was diagnosed by a physician, in 2 studies by a parent and in 1 study asthma was reported by adolescents. In 8 studies exposure was assessed according to BMI percentiles based on CDC growth charts, one study in which BMI-Z score was based on CDC growth chart, 4 studies in which BMI cut off point was based on IOTF, one study was based on reference data, one study was based on the BMI percentile values and one study was based on BMI- SDS units (z-score); also quality scores of the whole manuscript were good, see Table 1.

### Overall OR for overweight individuals

**Fig. 2:**
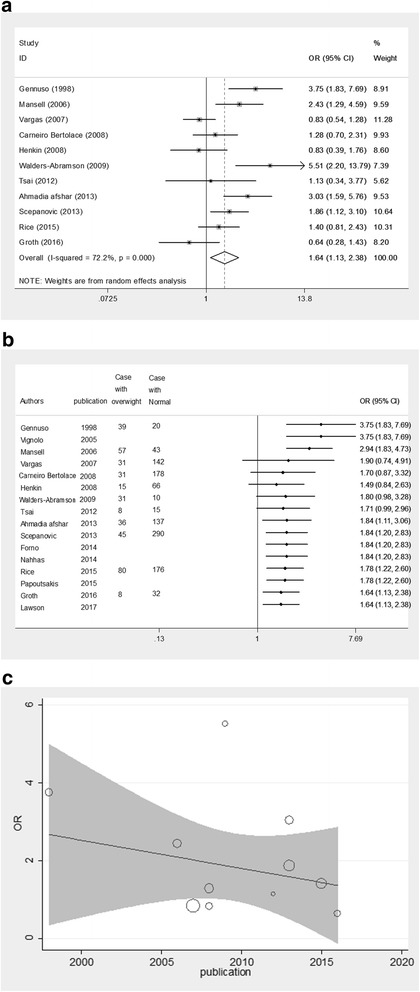
Meta-analysis based on 11 case-control studies which reported asthma in overweight individuals. **a** Forest plots of estimate of overall odds ratio asthmatic **b** cumulative meta-analysis and **c** meta-regression analysis with OR of asthma in overweight individual and year of publication

Meta-analysis derived OR = 1.64 (95% CI: 1.13–2.38, *p*-value = 0.01) from 11 case-control studies reported OR of asthma and overweight; moderate heterogeneity was observed between studies (Heterogeneity chi-squared = 35.91 (df = 10) *p*-value = 0.0001 and I^2^ = 72.2%) (Fig. 2a); However the reported relative risk was RR = 1.26 (95% CI: 1.07–1.48, p-value = 0.006). A cumulative meta-analysis showed that by combining the studies that were done before 2007, there was a significant association between overweight and asthma. By adding new studies from 2007 to 2012 to previous studies the cumulative effect of being overweight on asthma was not significant, while by adding studies that were done from 2013 to 2016 to previous studies, the cumulative effect of being overweight on asthma showed significant effects. (Fig. 2b). Meta-regression analysis showed that there was no significant statistical relationship between OR of asthma in overweight individuals and the year of publication. This means that the year of publication is not a reason for heterogeneity. (Correlation Coefficient = − 0.10, *p*-value = 0.260) (Fig. 2c).

### Overall OR for obesity

**Fig. 3:**
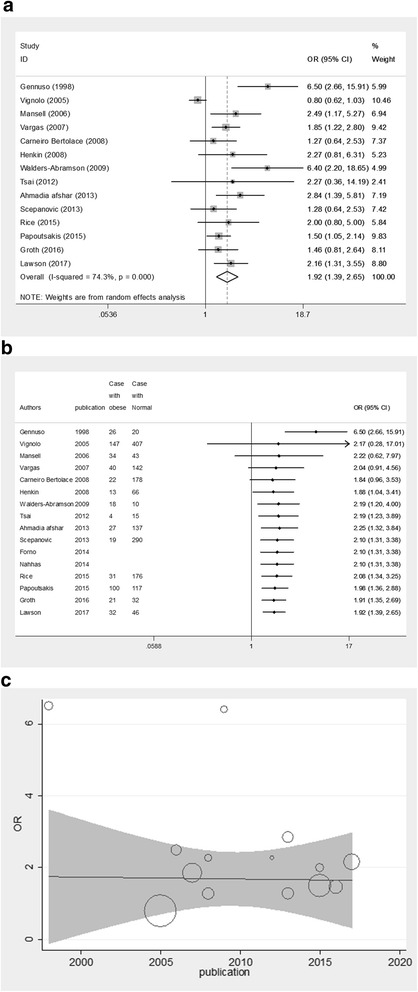
Meta-analysis based on 14 case-control studies which reported asthma in obesity individuals. **a** Forest plots of estimate of overall odds ratio asthmatic **b** cumulative meta-analysis and **c** meta-regression analysis with OR of asthma in obese individual and year of publication

In total, 14 case-control studies reported OR of asthma and obesity; meta-analysis revealed an association between them; OR = 1.92; (95% CI: 1.39–2.65, p-value = 0.0001). Furthermore, high heterogeneity was observed between studies (chi-squared = 50.49 (df = 13) *p*-value = 0.0001 and I2 = 74.3%) (Fig. 3a), as well as relative risk which was RR = 1.40; (95%CI: 1.19–1.63, *P*-value =0.0001). Cumulative meta-analysis showed that one study was done in 1998 with significant association between obesity and asthma. Adding new studies from 2005 to 2008 to previous studies showed that the cumulative effect of obesity on asthma was not significant. On the other hand, adding studies from decade of 2008 to 2017 to previous ones; a significant association was observed based on the cumulative effect of obesity on asthma (Fig. 3b). Meta-regression analysis didn’t identify any significant statistical relationship between OR for asthma in obese individuals and the year of publication. This means that the reason of heterogeneity is not the year of publication (Correlation Coefficient = − 0.15, p-value = 0.08) (Fig. 3c). Overall ES of asthma based on adjusted OR.

Three studies with 740 cases and 1169 controls were reported ES = 1.30 (95% CI: 1.12–1.49; *p*-value < 0.001), that confirm a significant increased risk of asthma for individuals with BMI greater than the 85th percentile.

### Overall SMD for asthma and BMI

The overall standard mean difference (SMD) based on 10 studies with 2761 cases, and 3281 controls, and the results showed a significant relationship between asthma and BMI (SMD = 0.21; 95% CI: 0.03–0.38; *p*-value = 0.021), for individuals with BMI greater than the 85th percentile.

### Subgroup analyses for asthma and overweight / obesity

Analysis showed that the risk of asthma in obese and overweight children during 2009–2017 was increased in comparison with the decade of 1998–2008. Furthermore, the risk of asthma in obese and overweight girls was greater than the risk of asthma in obese and overweight boys. Asian children and adolescents had a risk of asthmatic attacks three times more likely than children in the American continent (Table 2).

**Table 2:** The risk of asthma in obese and overweight children based on OR

Variables	Overweight			Obesity	
	Group	Study	OR (95% CI)	Study	OR (95% CI)
Year of publication	1998–2008	5	1.49 (0.84–2.63)	6	1.88 (1.04–3.41)
	2009–2017	6	1.80 (1.07–3.03)	8	1.90 (1.43–2.53)
Age	< 11 year	2	0.86 (0.58–1.29)	2	1.65 (1.26–2.15)
	> 12 year	3	1.30 (0.64–2.64)	3	1.61 (1.09–2.37)
	Mix	6	2.19 (1.35–3.55)	8	2.27 (1.26–4.06)
Gender	Male	2	1.46 (0.64–3.34)	2	1.14 (0.62–2.09)
	Female	2	1.51 (0.88–2.58)	2	1.65 (0.95–2.89)
Continents	America	9	1.50 (0.97–2.33)	10	2.21 (1.65–2.98)
	Europe	1	1.86 (1.12–3.10)	3	1.12 (0.70–1.79)
	Asia	1	3.03 (1.59–5.76)	1	2.84 (1.39–5.81)
Sample	< 500	8	2.00 (1.31–3.04)	8	2.71 (1.80–4.07)
	> 500	3	1.04 (0.56–1.93)	6	1.41 (0.98–2.03)
Report of Asthma	Physician	10	1.69 (1.11–2.56)	13	2.00 (1.42–2.82)
	Parent	1	1.28 (0.70–2.31)	1	1.27 (0.64–2.53)

### Subgroup analyses based on SMD

We found that there were significant relationships between BMI (greater than the 85th percentile) and asthma in a) both genders, b) results reported in 2009–2017, c) America and the Asian continent, d) children younger than 11 years old and e) groups of children whose asthma were reported by Physicians (Table 3) according to SMD.

**Table 3:** The risk of asthma in obese and overweight children based on SMD

Variables	Group	Study	SMD (95% CI) Overweight and Obesity
Year of publication	1998–2008	3	0.07 (−0.19–0.32)
	2009–2017	7	0.26 (0.08–0.45)
Age	< 11 year	3	0.40 (0.11–0.68)
	> 12 year	3	0.14 (−0.06–0.35)
	Mix	4	0.12 (−0.09–0.33)
Gender	Male	3	0.35 (0.25–0.44)
	Female	3	0.58 (0.19–0.98)
Continents	America	5	0.15 (0.03–0.26)
	Europe	3	0.06 (−0.15–0.26)
	Asia	2	0.52 (0.35–0.68)
Sample	< 500	4	0.27 (0.04–0.49)
	> 500	6	0.17 (−0.07–0.41)
Report of Asthma	Physician	8	0.17 (0.02–0.31)
	Parent	2	0.31 (−0.23–0.86)

### Bias

**Fig. 4:**
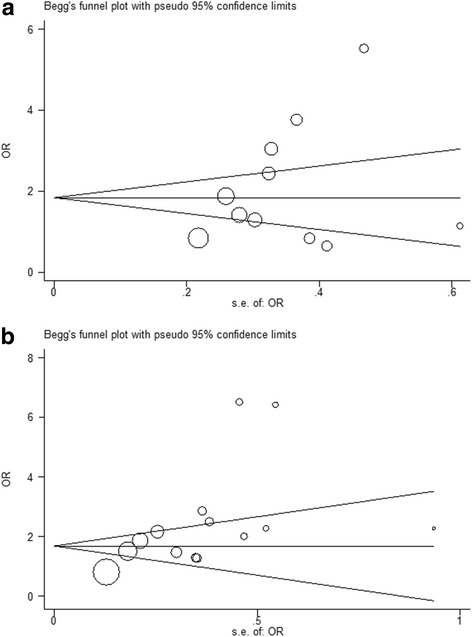
Begg’s funnel plot (pseudo 95% confidence limits) showings the effect of publication bias. **a** Overweight group and **b** Obese group

In order to evaluate the publication bias of studies, Begg’s test and the Funnel plot were employed. For articles related to overweight children, the *p*-value = 0.312 (Fig. 4a); for articles related to obesity the *p*-value =0.090 (Fig. 4b) and this identified that publication bias was not significant which shows that the majority of the query articles had the same opportunities to be published.

## Discussion

We determined that the risk of asthma in individuals who were overweight and obese was 1.64 times and 1.92 times more likely than individuals who were underweight/normal weight respectively. The ES obtained from a combination of adjusted the OR in BMI > 85 percentile was 1.30 (significant difference). Moreover, according to SMD, the relationship between asthma and BMI was significant. Beuther et al. noted that, based on the studies of animals, inflammation of airways due to allergic/non-allergic factors increased through the use of leptin from internal and external sources [36]. The relationship between obesity and asthma could be explained by a number of hypotheses, for example, obesity through hormonal influences or mechanisms of genetic factors which may have direct effects on immune system response or phenotype of asthma. Furthermore, an increased risk of asthma may be explained by a combination of genetic predisposition factors with birth weight, movement of the body that uses energy, and nutrition, as potentially linked to obesity [37]. So asthma is an outcome of a complicated combination of environmental and genetic factors for which we do not have thorough knowledge [38, 39]. However, the key point is that along with the increased risk of asthma with obesity, there are internal and external factors that count as confounders which might influence the relationship between asthma and BMI. The results from our meta-analyses showed a significant relationship between overweight/obesity and asthma, but the important result is that by removing the confounding factors, the effect size was reduced from 1.64 and 1.92 (in the overweight/obese) to 1.30. In this review, five studies reported adjusted OR according to different sets of confounding factors. The confounding factors in Henkin’s research were atopic dermatitis, allergic rhinitis, and other allergies [40]; in Papoutsakis’ research OR was adjusted for age, gender, education, atopic background of parents, calorie intake, breastfeeding, and physical activity score [41]; Forno et al. considered family income, the asthma record of parents, age, gender, and race to adjust the OR [42]; Also Nahhas considered the parents’ age, birth weight, education, smoker parents, physical activity, exposure to animals, watching TV, allergens, and gender as confounding factors [43]. Lawson et al. considered age, gender, mother’s education, having a asthmatic record in the family, early respiratory illness, smoker mother, smoking during pregnancy, dog at home in last 12 months, cleaning or playing in pens or corrals regularly and farm dwelling as factors to adjust the OR [44].

Analyses based on OR in the subgroups showed that overweight and obesity increased the risk of asthma in both genders (girls more than boys); however, the relationship was not significant, probably due to a small number of studies and sample size, so we need more studies dealing with a larger sample size to obtain more accurate results. Moreover, a significant association was identified between BMI and the risk of asthma in both genders (girls more than boys) based on SMD. The SMD is a calculated quantitative index based on the difference between the means in the case and control groups (continuous variable) which follows a normal distribution. This index has more precision than OR since OR is calculated on the basis of frequency of variables. In addition, the two articles that found significant SMD are different from the articles which found insignificant OR in terms of the exposure factor. Chen et al. reported that obese and overweight boys were at higher risk of asthma compared to girls [9] but the results of Egan et al. found a significant relationship between overweight and asthma in boys and obesity and asthma in girls [11].

Obesity is firmly connected to breathing disorders and influences the function of the lungs. In fact, the high percentage of excessive body fat compresses the lungs and limits the free air movement because of its mechanical effect on the airways via central body fat [45]. Gender, atopy, family history of asthma (non-modifiable), and obesity (one of the few modifiable) are risk factors for asthma [46]. Even though exercise has minimal impact on lung function in asthmatic children, it should still be recommended by health care providers [47]. The difference in risks by continent may indicate the effect of the environment or race on the hazard of asthma. In general, healthcare providers overseeing obese kids and wishing to control their asthma should consider interventions such as weight loss, physical activity, and normalization of nutrient levels. Monitoring of complications related to obesity with designed prospective and clinical trial studies should also be taken into account.

### Limitations

One of the main limitations of this research was the variety of methods used in reporting the results e.g., some studies reported M ± SD (mean ± standard deviation) and others reported OR. In addition, definitions of obesity and overweight were not consistent over different studies. The number of studies was also another limitation. Furthermore, meta-analysis for case-control studies cannot identify the causal-temporal relationships between BMI and asthma.

## Conclusion

Based on our findings, we noted that BMI is a significant factor when it comes to asthma. We found that obesity and being overweight increase the risk of asthma. A thorough investigation to recognize the confounding factors on the relationship between asthma and BMI is also important for future epidemiological research.

## Additional file

Additional file 1: PRISMA Checklist S1. (DOCX 66 kb)

## Abbreviations

BMI: Body mass index
CDC: Centers for Disease Control and Prevention
CI: Confidence interval
ES: Effect size
IOTF: International Obesity Task Force
ISSAC: The International Study of Asthma and Allergies in Childhood
JBI: Joanna Briggs Institute
MeSH: Medical subject headings
OR: Odds ratio
ORs: Odds ratios
PRISMA: Preferred Reporting Items for Systematic Reviews and Meta-analyses
RR: Relative risk
SD: Standard deviation
SDS: Standard deviation score
SMD: Standardized mean difference
